# An Extension of PPLS-DA for Classification and Comparison to Ordinary PLS-DA

**DOI:** 10.1371/journal.pone.0055267

**Published:** 2013-02-11

**Authors:** Anna Telaar, Kristian Hovde Liland, Dirk Repsilber, Gerd Nürnberg

**Affiliations:** 1 Institute for Genetics and Biometry, Department of Bioinformatics and Biomathematics, Leibniz Institute for Farm Animal Biology, Dummerstorf, Germany; 2 Department of Chemistry, Biotechnology and Food Science, Norwegian University of Life Sciences, Ås, Norway; Banner Alzheimer's Institute, United States of America

## Abstract

Classification studies are widely applied, e.g. in biomedical research to classify objects/patients into predefined groups. The goal is to find a classification function/rule which assigns each object/patient to a unique group with the greatest possible accuracy (classification error). Especially in gene expression experiments often a lot of variables (genes) are measured for only few objects/patients. A suitable approach is the well-known method PLS-DA, which searches for a transformation to a lower dimensional space. Resulting new components are linear combinations of the original variables. An advancement of PLS-DA leads to PPLS-DA, introducing a so called ‘power parameter’, which is maximized towards the correlation between the components and the group-membership. We introduce an extension of PPLS-DA for optimizing this power parameter towards the final aim, namely towards a minimal classification error. We compare this new extension with the original PPLS-DA and also with the ordinary PLS-DA using simulated and experimental datasets. For the investigated data sets with weak linear dependency between features/variables, no improvement is shown for PPLS-DA and for the extensions compared to PLS-DA. A very weak linear dependency, a low proportion of differentially expressed genes for simulated data, does not lead to an improvement of PPLS-DA over PLS-DA, but our extension shows a lower prediction error. On the contrary, for the data set with strong between-feature collinearity and a low proportion of differentially expressed genes and a large total number of genes, the prediction error of PPLS-DA and the extensions is clearly lower than for PLS-DA. Moreover we compare these prediction results with results of support vector machines with linear kernel and linear discriminant analysis.

## Introduction

In discrimination studies, data sets are often handled having high numbers of features but only few samples. Especially for gene expression experiments, where thousands of genes are measured and in comparison only few samples are used, dimension reduction is advantageous as a pre-processing step before the final classification step takes place. There exist a lot of feature extraction methods for dimension reduction. One such method is powered partial least squares discriminant analysis (PPLS-DA) which is a specialized version of the well-known partial least squares discriminant analysis (PLS-DA), which was first introduced in chemometrics by Wold et al. [1] by using the PLS regression [2] for classification purposes. Here the response variable (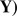 in the linear model is given in form of indicator variables. Barker & Rayens [3] and Nocairi et al. [4] were the first to formulate PLS-DA accurately. The aim is to reduce the dimensions (number of features) by coordinate transformation to a lower dimensional space. PPLS-DA was introduced by Liland and Indahl in [5] to improve the calculation of the loading weights for better separation of the groups by introducing a power parameter analogously to powered partial least squares (PPLS) [6] and maximizing the correlation between the data matrix and the group memberships, analogous to Fisher’s canonical discriminant analysis (FCDA). The optimization criterion in PPLS-DA is therewith not directly aimed at prediction, and therefore the original algorithm does not necessarily yield the best components for class prediction.

Former studies of Telaar et al. in [7] show similar prediction errors (PEs) for PLS-DA and PPLS-DA for most of the analysed data sets, and even lower error rates compared to other classification methods e.g. random forest and support vector machine. Therefore, we try to optimize the power parameter of PPLS-DA towards class prediction in a training set, to see if the prediction result for a test set can be improved. The power parameter and the number of components are determined according to the lowest prediction error of a linear discriminant analysis (LDA) using the PPLS-DA components, taking a cross-validation approach. Furthermore, we compare the results of this extension and of PPLS-DA with the ordinary PLS-DA with respect to prediction error (PE) for simulated data sets and five publicly available experimental data sets. Finally the PPLS-DA results of the simulated and experimental data sets are compared to those of support vector machine (SVM) with linear kernel and LDA with ten features selected according to the t-test.

## Materials and Methods

### Description of a Classification Problem

An 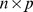 data matrix 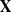 with 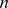 objects and 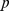 features together with a vector 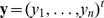 of group memberships form the basis of a classification problem. Here 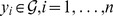 is the group label for object 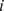 which for example can be given in the form of discrete variables or symbolics. The k-th column of the matrix 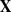 is denoted by 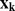. We assume that each sample belongs to a unique group 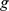 and each group has a sample size of 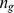 and a prior probability of group membership of 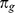. In total 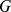 different groups exist and 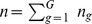. In this article, we restrict ourselves to only two different groups 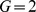. Our results can be extended to more than two groups.

The group information can also be given in the form of a dummy coded matrix 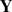 as follows 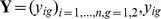 equals 1 if sample 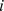 belongs to groups 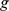, otherwise the entry equals 0, 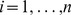 and 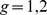. The goal is to determine a function 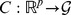 which assigns to each object a unique group (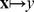) with the greatest possible accuracy [8]. In the following we assume that 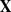 is centered. Therewith the empirical total covariance matrix 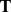 is equal to 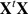, and let 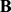 denote the empirical between group sum of squares and cross-product matrix, which can be formalized as 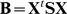 with 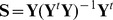 [8].

### Introduction of PLS-DA and PPLS

#### PLS-DA

Before we start with the detailed description of PPLS-DA, we give a brief introduction to the roots of this method, PLS-DA and PPLS. Let 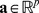 be a vector of loadings with 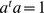 and 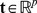 a vector containing the mean values of the groups, 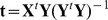. Nocairi et al. [4] showed that the dominant eigenvector of 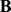 maximized the covariance between 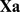 and 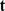 in the context of classification. Therefore this eigenvector should be used for the determination of the loading weights vector which was also recommended by Barker & Rayens [3]. An enhanced version of PLS-DA with inclusion of prior probabilities (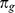) in the estimation of 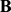 is proposed by Indahl et al. [8]. In this version, the importance of each group no longer depends on the empirical prior probabilities. Therewith a direct opportunity is given to put more weight on special groups for the calculation of the loading weights. Moreover 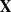 is transformed to an 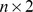 matrix 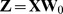, here 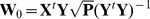 and 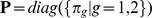. Therewith a 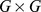 eigenvalue problem (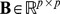) is reduced to a 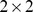 eigenvalue problem (2 = number of groups), because only the between-group covariance matrix according to 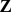 is considered (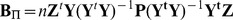). Afterwards the eigenvalue needs to be back transformed by 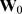.

#### PPLS

Now we are turning back to PLS regression, to explain the introduction of the power parameter 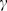 in PLS regression. The loading weights vector of PLS regression 
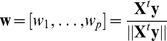
 maximizes 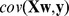 and can be rewritten as.
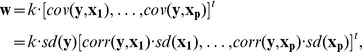
with a scaling constant 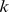 where 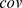, 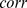 and 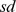 denote covariance, correlation and standard deviation, respectively. Hence, the influence on the loading weights of the correlation and standard deviation part is balanced. Dominating 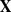-variance which is irrelevant for prediction does not lead to optimal models, therefore Indahl et al. [6] propose PPLS which allows the user to control the importance of the correlation part and the standard deviation part by a power parameter 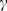 as follows:



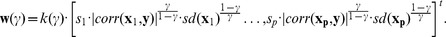



Here 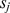 denotes the sign of 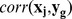. The power parameter 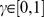 is determined such that the correlation is maximal: 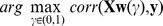. Also 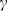 equal to 0 and 1 is included in the maximization problem by calculation correlation with a loading weights vector which has only a non-zero entry (for 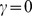) for the feature with the largest standard deviation and by determining the correlation according to a loading weights vector which has only a non-zero entry for the feature with the largest correlation to 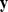 (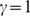).

### PPLS-DA: Optimization of the Power Parameter According to Correlation

PPLS-DA is designed to deliver components which are optimal for discriminating the cases coded in 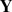. This optimality can be understood in terms of a correlation approach. Nocairi et al. showed in [4], that correlation is determined by the so called Rayleigh quotient.
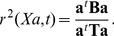
(1)


Here the correlation is measured by the squared coefficient of correlation 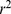. Maximization of the correlation is therewith equivalent to maximization of the Rayleigh quotient.
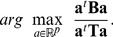
(2)


The well known solution of the maximization problem (2) is the dominant eigenvector 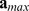 of 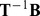. This is exactly the approach of Fishers`s canonical discriminant analysis (FCDA) for determining the vector of loadings.

For PPLS-DA, Liland and Indahl combine in [5] the approaches of FCDA and PPLS and further include, like in [8], prior probabilities 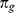 for each group 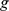 in the calculation of 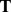 and 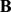. The data matrix 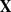 is transformed with 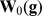 to the 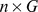 matrix 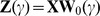, where 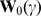 contains the possible candidate loading weights vectors as columns.
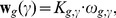
(3)


with 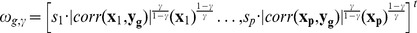,


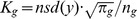
, 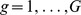 and 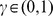.

The power parameter 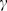 enables the focus on features which have a high correlation to 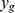 or on features which have a high standard deviation. For the transformed matrix 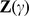 the between group sum of squares and cross-product matrix including prior probabilities can be calculated as follows 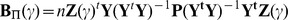 and the total variance matrix is obtained as 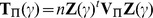 with 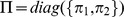 and 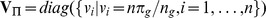 [5]. 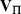 is an 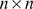 diagonal matrix where the non-zero entry belonging to sample 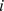 is the ratio of the prior probability of the group to which 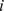 belongs and the corresponding group size times the number of samples 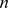.

The maximization problem of PPLS-DA is as follows.
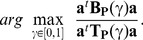
(4)


For 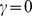 the feature(s) with the highest standard deviation are tested and for 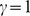 the feature(s) with the highest correlation to the group membership vector are checked separately. We denote the solution of the optimization problem (4) by 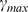. To avoid singular matrices 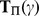 and to get a numerically more stable solution, Liland and Indahl [5] substitute the maximization problem (4) by the maximization problem 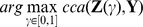, where cca denotes the canonical correlation. In [9] the authors showed that these procedures lead to equal results.

For each component, 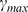 is determined by maximization of the canonical correlation:
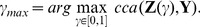
(5)


In the algorithm of PPLS-DA in the R package pls, the R function *optimize* is used to search for the maximum. For this purpose, the whole training set is used to find 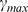. Transforming 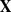 into an 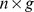 data matrix 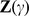, the maximization problem depends on the number of groups and number of samples. It does not depend on the usually much higher number of features. The final loading weight vector is then 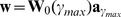. For a detailed description of PPLS-DA we refer to [5].

In our experience with PPLS-DA, we have observed that optimization of 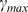 does not always lead to the lowest possible prediction error available through other choices of 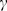.

### PPLS-DA: Optimizing the Power Parameter 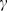 with Respect to Prediction

To improve prediction errors in classification tasks using PPLS-DA, we aim at optimizing the power parameter 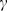 with respect to prediction error of LDA. For this, we propose a cross-validation approach, to avoid overfitting. Therefore, we separate in a first step the data into a training and a test set which are disjunct. We now call these sets outer training set and outer test set. Using only the outer training set to optimize the 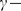
value, we use the outer test set to evaluate the computed classification function. In a next step we split the outer training set randomly in 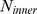 different inner training and inner test sets. Because unbalanced data have influence on the estimated classifier, we down-sample the majority group objects to get equal numbers of objects in both groups for the outer training set. Here, the proportion of 0.7 of the smallest group size determines the size of the outer training set for each group. The remaining objects build the outer test set. For the optimization step, we take into account equidistant fixed 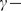
values in [0,1] with step size 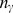, resulting in a sequence of 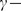
values for the optimization 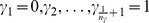. For example for a choice of 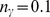, we consider 11 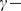
values, 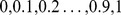.

**Figure 1:**
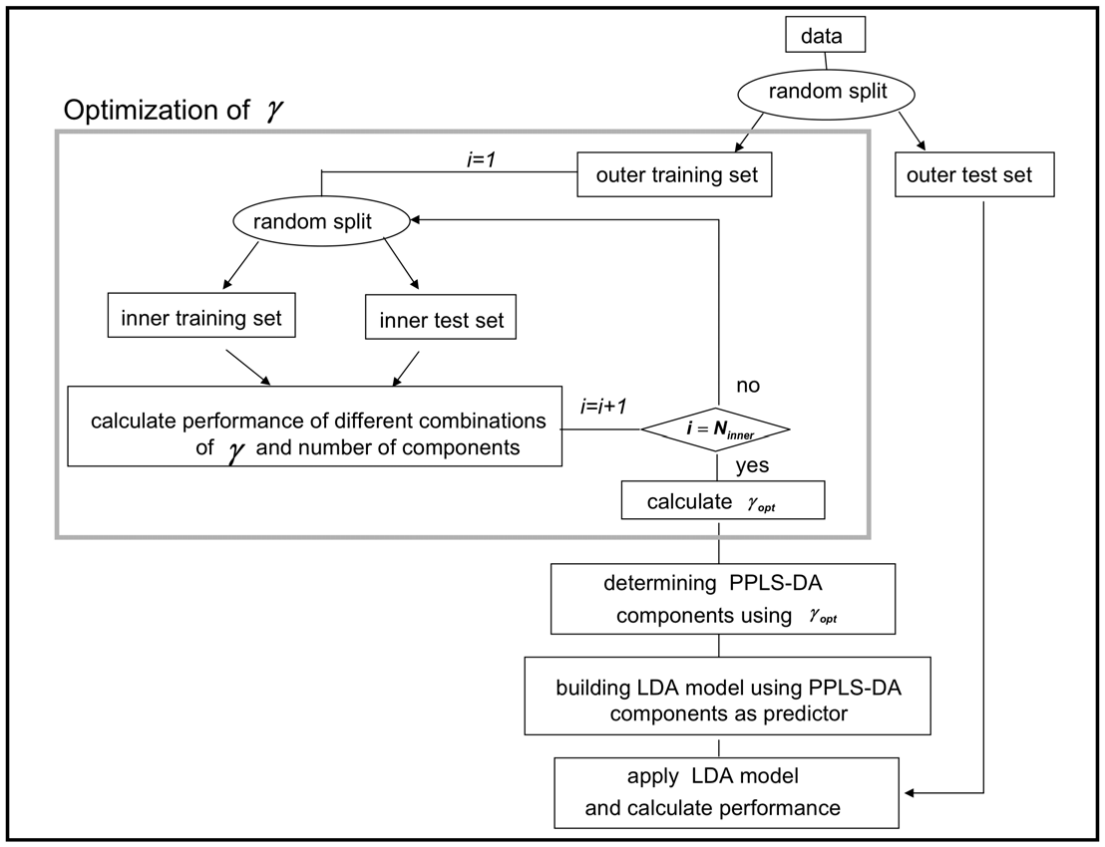
Rough overview of the proposed extension of PPLS-DA.

We calculate the prediction error (PE), the proportion of wrongly classified samples of a test set, as a measure for good classification. In Figure 1 a rough overview of our proposed extension is given. In this paper, all cross-validation procedures consist of random samples of the corresponding data sets to the proportions of 0.7 (training set) and 0.3 (test set). For example, a cross-validation with 10 repeats, repeats the sampling 10 times. Utilizing the statistical software R, we use the function cppls (of the R-package pls) for PPLS-DA and the function lda (of the R-package MASS). Furthermore, we use the default setting for the priors in the lda function, using the proportions of the groups which are equal in our cases.

We propose an extension for optimizing both, 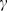 and the number of components to be used as input for the LDA. The optimization of the 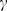-value depends on the parameters 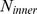 and 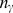.

In our extension all components are used to optimize the 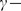
value minimizing PE. The number of components and the power parameter are optimized in one procedure. Therewith all components have the same 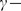
value.

**Figure 2:**
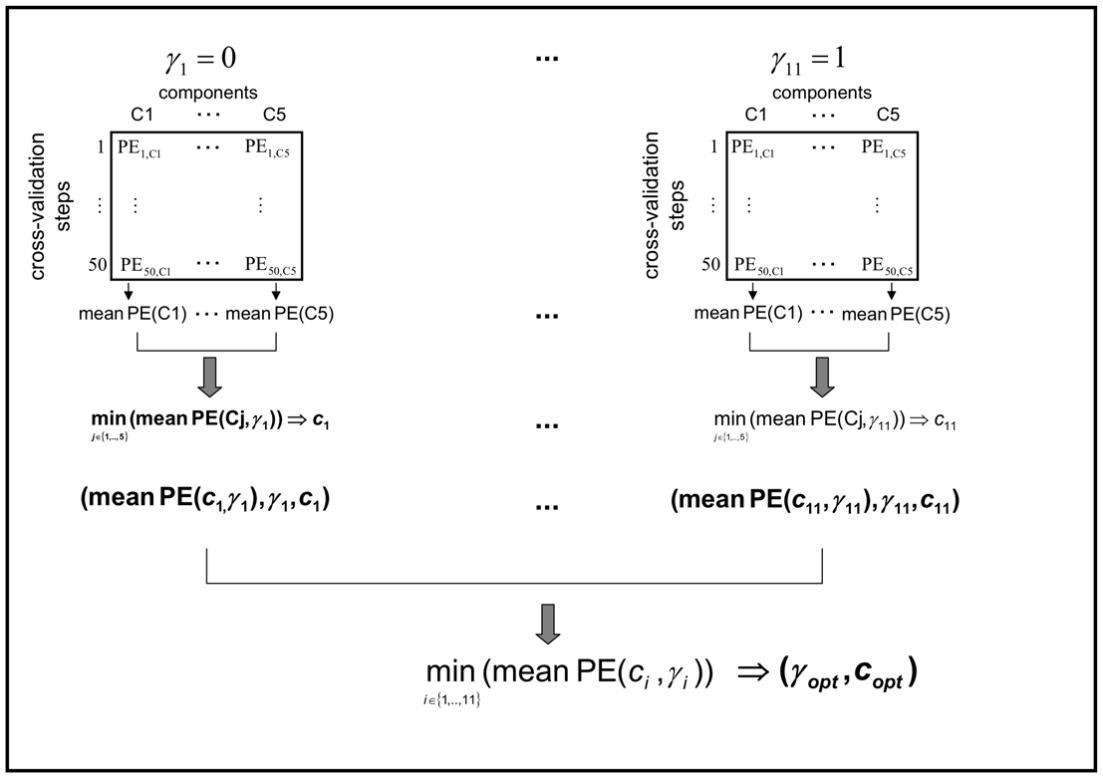
Extension of PPLS-DA - for stepsize 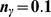 and 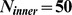. The power parameter is denoted by 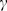, the prediction error (number of wrongly classified samples of the inner test set) is abbreviated with PE. 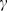 varied in 11 steps (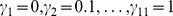). Cj, j = 1
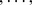
5 is short for the jth component. The function min(f) takes the minimum of function 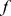. The cross-validation procedures consist of random samples of the outer training set to the proportions of 0.7 (training set) and 0.3 (test set). The cross-validation steps are conform to sampling with replacement. The optimal 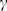-value and the optimal number of components are determined after 50 repeats.

In the optimization, for each fixed 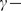
value (
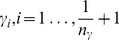
) the optimal number of components of PPLS-DA is determined as follows (see Figure 2): For the 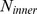 different inner test sets, the PE for one up to five components Cj, j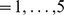 is calculated, all using the same 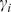 resulting in a matrix 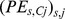, 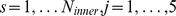. Then the average inner PE is calculated for each component. Therewith we select for each 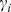 the smallest mean PE over the 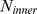-test sets with a corresponding optimal number of components (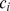). We search for the minimal PE leading to 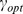 with the optimal number of components 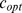. For these 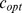 components and the power parameter 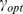, we calculate the corresponding loading weights vectors on the outer training set, and we finally determine the PE of the outer test set.

### R Functions Used

#### (P)PLS-DA implementations

For PPLS-DA, we used the R-function cppls of the R-package pls. We implemented an R-code for PLS-DA based on [8]. The optimal number of components was determined by a cross-validation on the outer training set for PLS-DA, PPLS-DA with 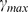 and our described extensions of PPLS-DA. For this step, we restricted the maximal number of components to five. The segments of the cross-validation are randomly chosen at the proportion of 0.7 and 0.3 of the data set. We repeat this procedure 10 times.

### Further Classification Methods

#### SVM

The classification method SVM with a linear kernel searching for a linear hyperplane for the separation of the data is considered for comparison. For this purpose the R package e1071 [10] is applied and the parameter 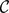 for the linear kernel is tuned within the interval 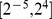 using the R-function tune.svm with a cross-validation of 10 steps. The interval for tuning is chosen according to the suggestion of Dettling & Buehlmann [11].

#### t-LDA

Additionally an LDA is performed using ten features which are filtered based on the outer training set according to a ranking list based on the lowest p-value of the t-test. For the t-test we use the R-package stats.

The same segments of the outer training and outer test set are used across all tested methods for fair comparison.

### Data

We investigate simulated data and five publicly available experimental data sets. After preprocessing (like mentioned in the description of the experimental data sets), all experimental data are on the log-scale of gene expression as for the simulated data.

**Figure 3:**
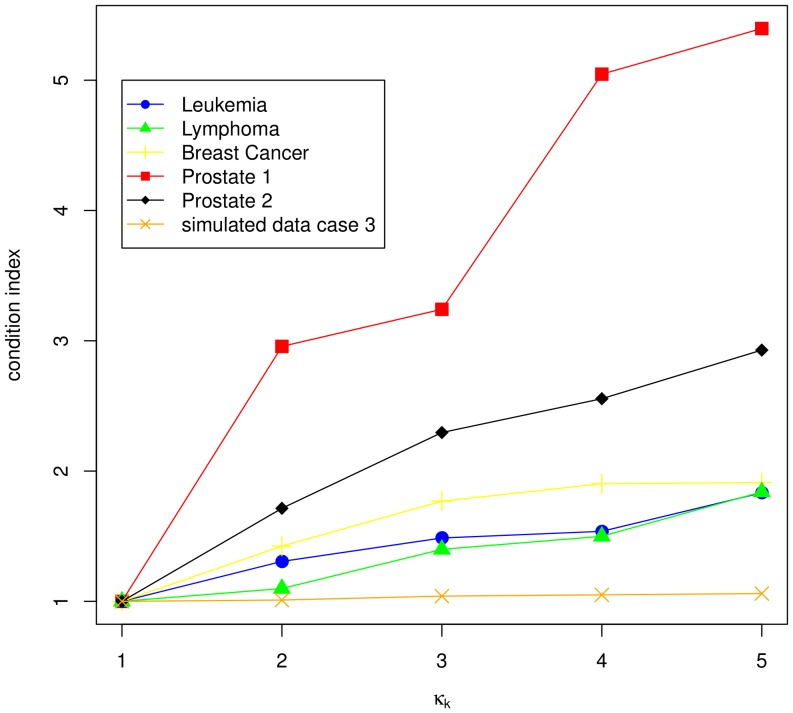
Condition index for the first five eigenvalues. The condition index 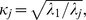 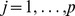 (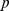 number of features) is used as a measure for variable dependence, with 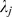 eigenvalue of 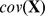. It can be assumed that 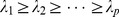. The increase of the first five condition indexes (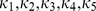) reflects the collinearity of the features. A rapid increase means, the features are strong linear dependent, a weak increase implies a weak dependence.

**Figure 4:**
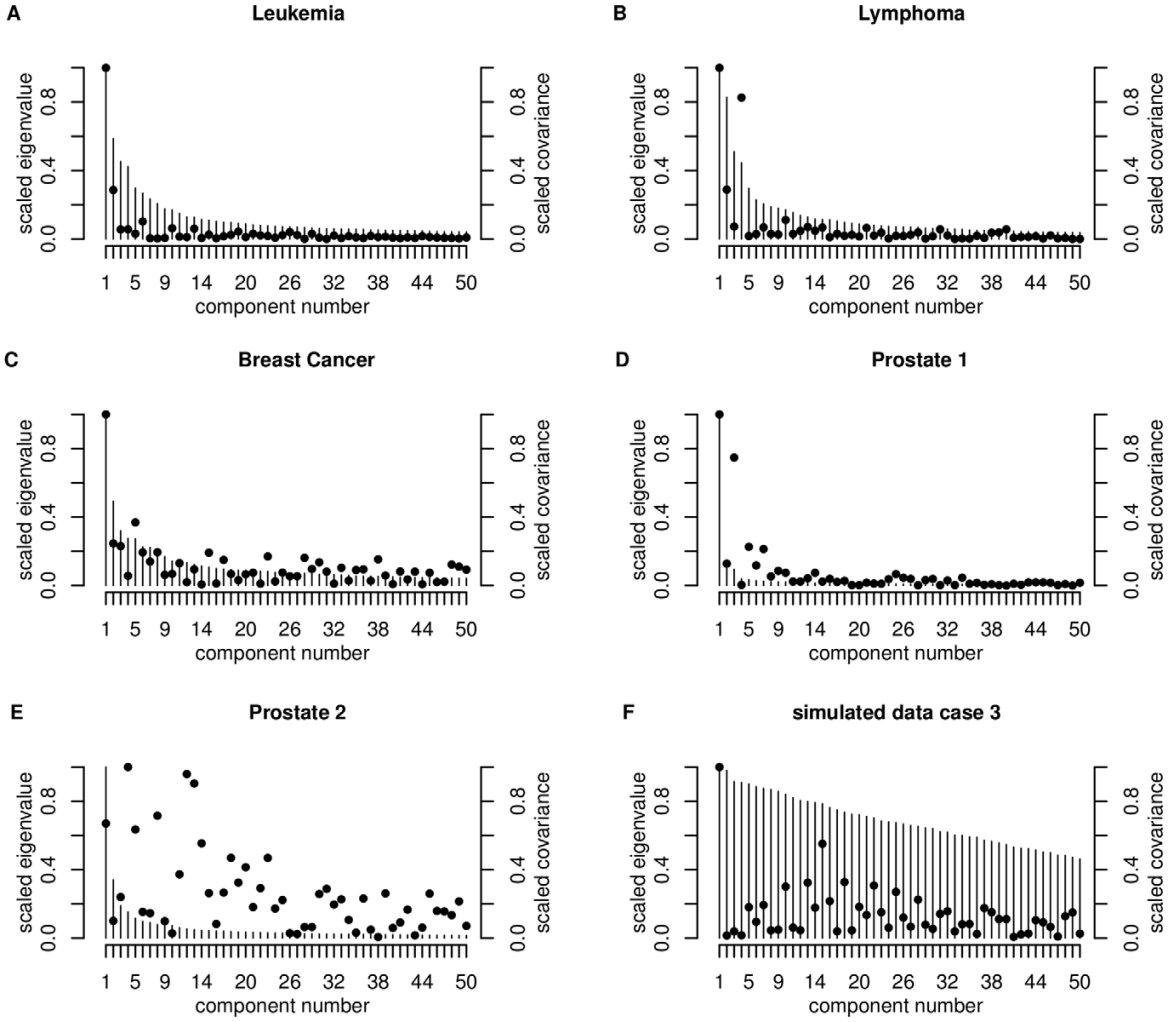
Plot of the first 50 largest eigenvalues 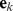 of cov(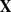) (bars) and of the absolute covariance between 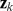 and 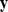 (dots) for the experimental data sets and for case 3 for the simulated data. The eigenvalues 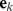, 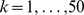 are scaled corresponding to the largest eigenvalue, also the absolute values of the covariance between the principal component 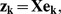 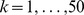 and the response vector 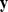, here 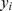 equals 1 if sample i belongs to group 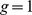, otherwise 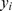 equals −1.

For detailed description of the covariance structure of our data, we use two measures analogous to Sæbø et al. in [12]. The condition index, first used in [13], and the absolute value of the covariances between the principal components of 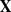 and the response vector as used in [12]. The condition index 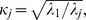 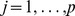 is used as a measure for variable dependence, with 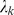 being the kth eigenvalue of 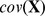. It can be assumed that 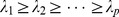. The increase of the first five condition indexes (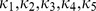) reflects the collinearity of the features. A rapid increase means, the features have a strong linear dependence, a weak increase implies a weak dependence. If we now consider the principal components, like in [12], the relevance of a component is measured by means of the absolute value of the covariances (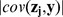) between the principal component 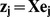 and the class vector 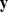. Here 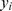 equals 1 if sample 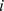 belongs to group 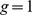, otherwise 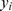 equals -1, 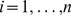. The eigenvector belonging to the kth largest eigenvalue is denoted by 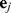. Helland and Almøy [14] infer, that data sets with relevant components, which have small eigenvalues, are difficult to predict. The condition index is plotted for the first five largest eigenvalues (scaled to the first eigenvalue) in Figure 3. Figure 4 shows the first 50 largest scaled eigenvalues and the corresponding scaled covariances between 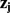 and 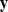 for all experimental data sets and a simulated data set (case 3) investigated.

#### Simulated data sets

Gene expression data are simulated as normally distributed data, considering the log scale of microarray intensities after normalization: 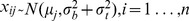 and 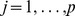. Here 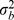 denotes the biological variance which we chose equal to 0.04, and 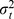 represents the technical variance which we chose in different proportions of the biological variance. Studying a two-class classification objective, we simulate 60 samples per class for the whole data set and 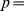
1000 genes partitioned in an informative part and non-informative part for the classification. For the test set, 30 single samples per class are randomly chosen. The remaining 30 single samples per class constitute the training set. The non-informative part of the data matrix which shows no differences between the two classes, consists of normally distributed random variables with mean 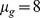 and biological variance 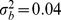, as well as different cases of technical variance 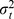. The informative part contains ten differentially expressed genes (DEGs) with a mean class difference 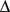 [7]. We take 5 cases into account. For case 1, 2 and 3, for each DEGs, 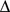 is chosen according to the uniform distribution from the interval 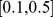. Case 1 has a technical variance of zero, case 2 of one-quarter of the biological variance (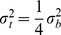), and case 3 is simulated with a technical variance of the same size as the biological variance (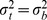). The ten DEGs of case 4 have a mean class difference of 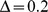 and 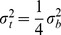. The simulated data of case 5 also have the high noise level (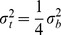), but a higher mean class difference with 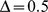.

We illustrate the data structure for the simulated data on the example of case 3. The condition indexes are 1.00, 1.01, 1.04, 1.05, 1.06. The increase is the weakest for all data sets considered (Figure 3), and therewith the genes are only weakly linear dependent, which is also shown by Figure 4F. This is similar for all simulated data cases. Furthermore, the proportion of DEGs is very low with 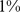 for all cases.

#### Experimental data sets

Additionally, we considered five publicly available experimental microarray gene expression data sets which are summarized in Table 1 containing information about the group size, number of genes, proportion of differentially expressed genes and original publication. For the determination of the number of differentially expressed genes (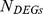) we use a t-test (from the R-package stats) and an FDR correction [15] (R-package qvalue). We count all genes with a q-value below 0.05. In the following the five data sets are described:

**Table 1:** Overview of the experimental data sets.

name	number of samples	number of genes	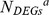	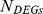 in %	original publication
Leukemia	47/25	3571	1445	40.46	[19]
Lymphoma	58/19	7129	1739	24.39	[20]
Breast cancer	44/34	4997	54	1.08	[17]
Prostate 1	50/52	6033	2393	35.26	[21]
Prostate 2	41/62	42129	595	1.40	[22]

a For the determination of the number of differentially expressed genes (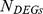) we use a t-test (from the R-package stats) and an FDR correction [15] (R-package qvalue). We count all genes with a q-value below 0.05.

The Leukemia data were downloaded from the Whitehead Institute website. We merge the training set and the test set to get a higher sample size and sample from these to get new proportions 0.7 and 0.3 for the training and test set. The R code for data preprocessing from http://svitsrv25.epfl.ch/R-doc/library/multtest/doc/golub.R is used which is according to [16]. The data set consists of two groups, 25 patients with acute myeloid leukemia and 47 patients with acute lymphoblastic leukemia and 3571 genes.

The condition indexes show a weak increase for this data set (1.00, 1.31, 1.49, 1.537, 1.83). This and the plot of the eigenvalues (Figure 4A) lead to the assumption of a weak linear dependency between the genes. The more relevant components have the largest eigenvalues (Figure 4A). Therefore we can expect good prediction performance of this data set. This data set has the highest proportion of DEGs (40.46%, Table 1).

The Lymphoma data set was downloaded from the website http://www.broadinstitute.org/mpr/lymphoma/. The data are GC-RMA normalized. Two groups are considered, 58 patients with diffuse large B-cell lymphomas and 19 patients with B-cell lymphoma, follicular lymphoma. Only genes with a non-zero variance are used in our analysis, which leads to 7129 genes.

The between-variable dependencies are comparable to the Leukemia data set (condition indexes: 1.00, 1.10, 1.40, 1.50, 1.84). The covariance structure (Figure 4B) is also comparable to those of the Leukemia data set and the total number of DEGs is a little bit higher than for the Leukemia data set, but the proportion on the total number of genes is clearly lower (24.3%, Table 1).

The Breast Cancer data set consists of normalized and filtered data, downloaded from http://homes.dsi. unimi.it/∼valenti/DATA/MICROARRAY-DATA/R-code/Do-Veer-data.R. The normalization was performed according to [17]. In this data set, only the two groups with the highest sample size are included: 34 patients with distant metastases within 5 years and 44 patients without, after at least 5 years. The total number of genes is 4997.

We found again a weak increase in the condition indexes for the first five eigenvalues (1.00, 1.42, 1.77, 1.90 and 1.91), but slightly faster than for the Leukemia and Lymphoma data set (Figure 3). The eigenvalue plot (Figure 4C) illustrates also a weak linear dependence between the features. The proportion of DEGs is the lowest for all experimental data sets (1.08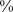, Table 1).

The Prostate 1 data set contains 52 tumor and 50 non-tumor cases and was downloaded from http://stat.ethz.ch/~dettling/bagboost.html. The preprocessing is described in [18] and the final data set contains 6033 genes.

This data set shows a rapid increase of the condition index from 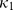 to 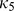 (1.00, 2.96, 3.24, 5.046, 5.397), describing a strong linear dependency of the genes (Figure 3). This property is also indicated by the plot of the eigenvectors (Figure 4D). This data set also has a high proportion of DEGs (32.26%, Table 1).

We downloaded the Prostate 2 data set, which was already normalized, from http://bioinformatics. mdanderson.org/TailRank/. A description of the normalization can be found at http://bioinformatics.mda nderson.org/TailRank/tolstoy-new.pdf. In this data set, only the two groups with 41 patients with normal prostate tissue and the 62 patients with primary tumors are in included.

The condition index shows a rapid increase (1.00, 1.71, 2.10, 2.56, 2.93) for the first five eigenvalues (Figure 3), but more moderate than for the Prostate 1 data set. Figure 4E shows that also relevant components have small eigenvalues, which indicates low prediction performance. The proportion of DEGs is very low (1.4%, Table 1) and similar to those of the Breast Cancer data set, but the total number of genes is the largest (42129) for all experimental data sets.

## Results

Results for the simulated data are based on 100 repeated simulations, and for the experimental data 100 different outer training and outer test sets are sampled. We present mean PE values of the outer test sets and the corresponding 95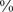 confidence intervals. At first, we describe and compare the results of PPLS-DA using 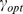 with PPLS-DA using 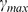, followed by the comparison between PLS-DA, PPLS-DA using 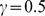, t-LDA and SVM.

We calculate confidence intervals for PE as follows: let 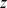 be the vector with 100 estimates of prediction errors. The upper bound of the confidence interval is then calculated as 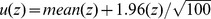. The lower bound is calculated likewise. If these confidence intervals overlap, we report no significant differences, if they are disjunct, the corresponding PEs are reported as significantly different.

### Results for the Simulated Data

#### PE results

At first we study the dependency of 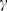 optimization on the step size 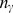, and on the number of internal cross-validation steps 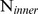. We calculate the mean PE results for 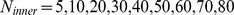 and 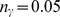 or 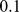. Because the corresponding 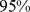 confidence intervals overlap for all different parameter choices for fix 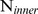 (Figure S1), we show all further results for 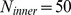 and 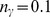.

**Figure 5:**
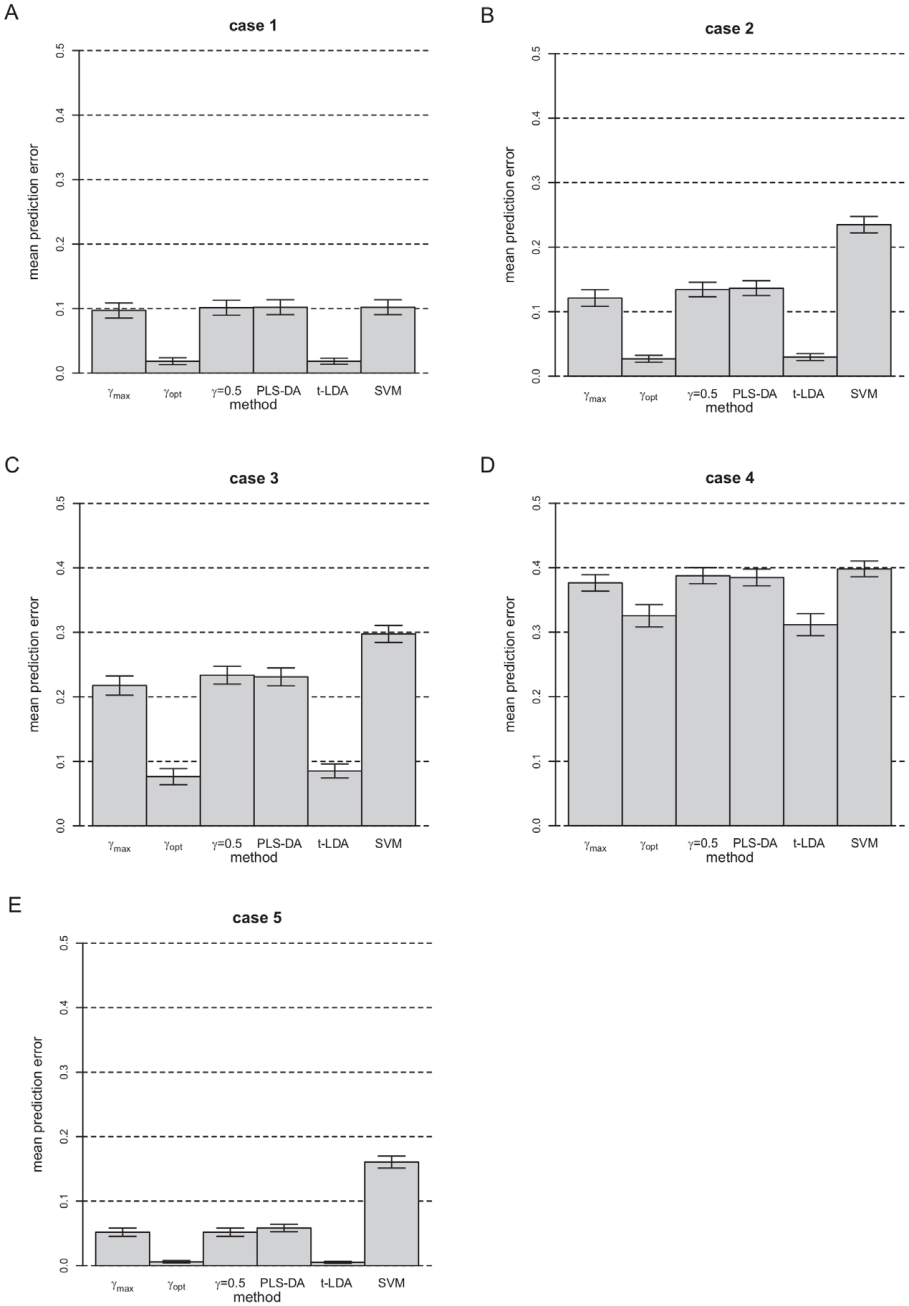
Mean PE of PPLS-DA using 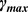, 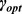 and 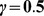, PLS-DA, t-LDA and SVM for the five cases of the simulated data.

Figure 5 illustrates the mean PE results and corresponding 95% confidence intervals for the simulated data and Table 2 summarizes the average number of components used for PLS-DA, PPLS-DA using 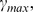 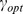 and 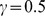.

**Table 2:** The mean number of components used for simulated data for 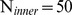 and 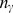 = 0.1.

simulated data			PPLS-DA with			PLS-DA
case	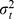	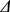	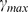	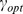	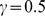	
1	0	[0.1,0.5]	2.6	1.9	2.7	2.7
2	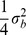	[0.1,0.5]	2.8	2.0	2.7	2.9
3	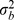	[0.1,0.5]	2.9	2.0	2.8	2.9
4	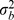	0.2	2.7	2.5	2.7	2.7
5	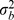	0.5	2.4	1.7	2.5	2.5

For all considered cases, PPLS-DA using 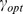 shows a significantly smaller PE than PPLS-DA using 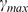. Especially for DEGs with 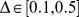, the PE of PPLS-DA with 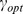 is only one-tenth of the PE for the PPLS-DA with 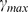 in the case without noise, one-fifth for a minor noise level (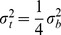) and still one-third for a high noise level with 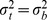.

Considering the frequency distributions of 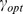-values (Figure S2 shows the corresponding histograms for case 3), the value with the highest frequency for the optimal 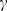-value (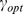) determined in a cross-validation approach is 0.8. This is in contrast to the values for 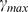, with 0.5 as highest frequency (Figures S2A and B, see Supplementary Materials).

**Figure 6:**
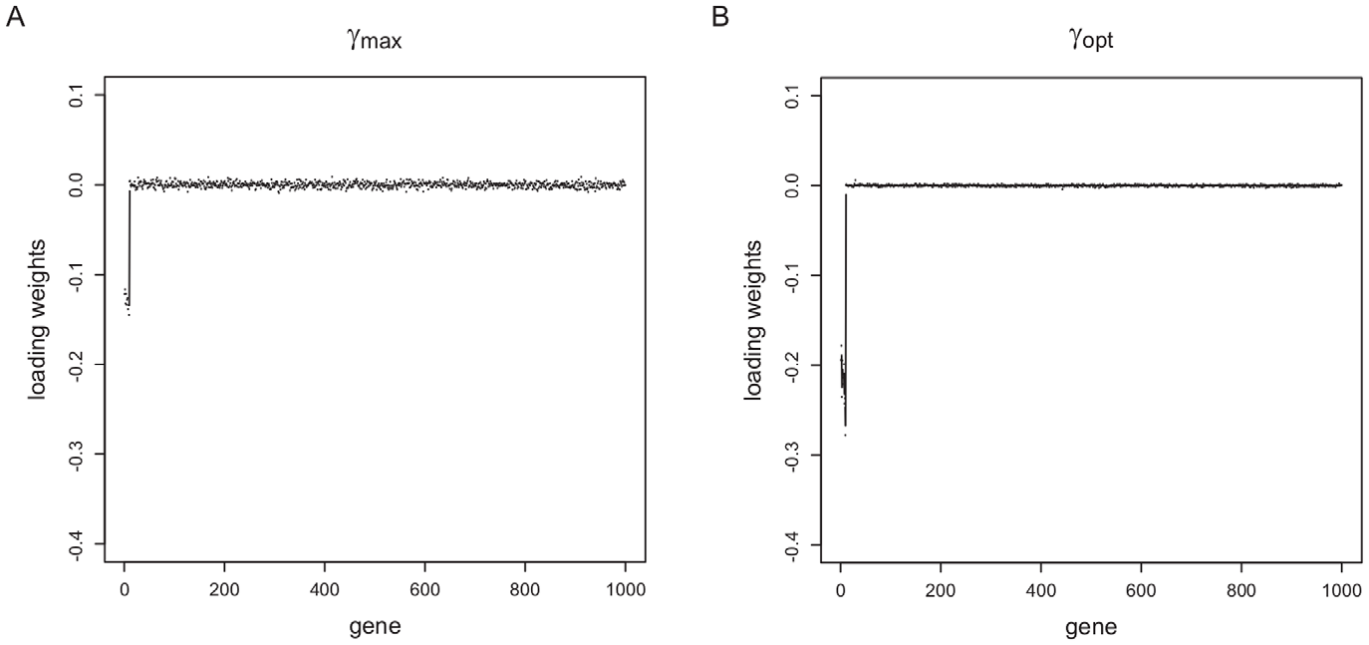
Average loading weights of the first component for the simulated data (case 3). The simulated data of case 1 are constructed such that the technical variance is of the same size as the biological variance. 10 differentially expressed genes with a mean class difference 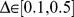 are simulated. Loading weights for the first component as calculated by PPLS-DA are shown with the power parameter 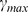 (A) and 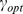 (B) using 50 inner cross-validation steps and a stepsize of 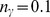. The basis are the results of 100 choices of the outer training and outer test set.

Different power parameters have large effects on the loading weights, which can be seen in Figure 6, e.g. for the first component for 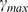 and 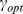 with 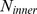 = 50 and 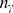 = 0.1 for case 3. The first 10 genes, which are simulated as differentially expressed, receive the highest absolute loading weight values for all methods. For 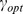, these loading weights of the informative genes are increasing in absolute values, and especially the non-informative genes receive loading weight values near to zero in comparison to the loading weights induced by 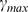.

Comparing our above findings of the PE with those of the PE of PLS-DA and of PPLS-DA with 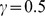, PLS-DA shows an equal PE to PPLS-DA using 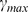 or 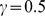, for all cases of the simulated data. Therewith also PPLS-DA using 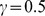 shows a significantly lower PE than PLS-DA. The number of components used for PLS-DA is equal to the corresponding number of components used for PPLS-DA using 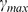 or 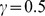. Hence overall, PPLS-DA using 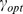 uses in average the lowest number of components.

Now, we consider the results of the two methods, SVM and t-LDA. For all simulated data cases, the method SVM shows the largest PE for all simulated data cases. The method t-LDA does not show a significant different PE to PPLS-DA using 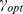.

### Results for the Experimental Data Sets

As for the simulated data, we choose 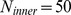 and 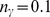.

**Figure 7:**
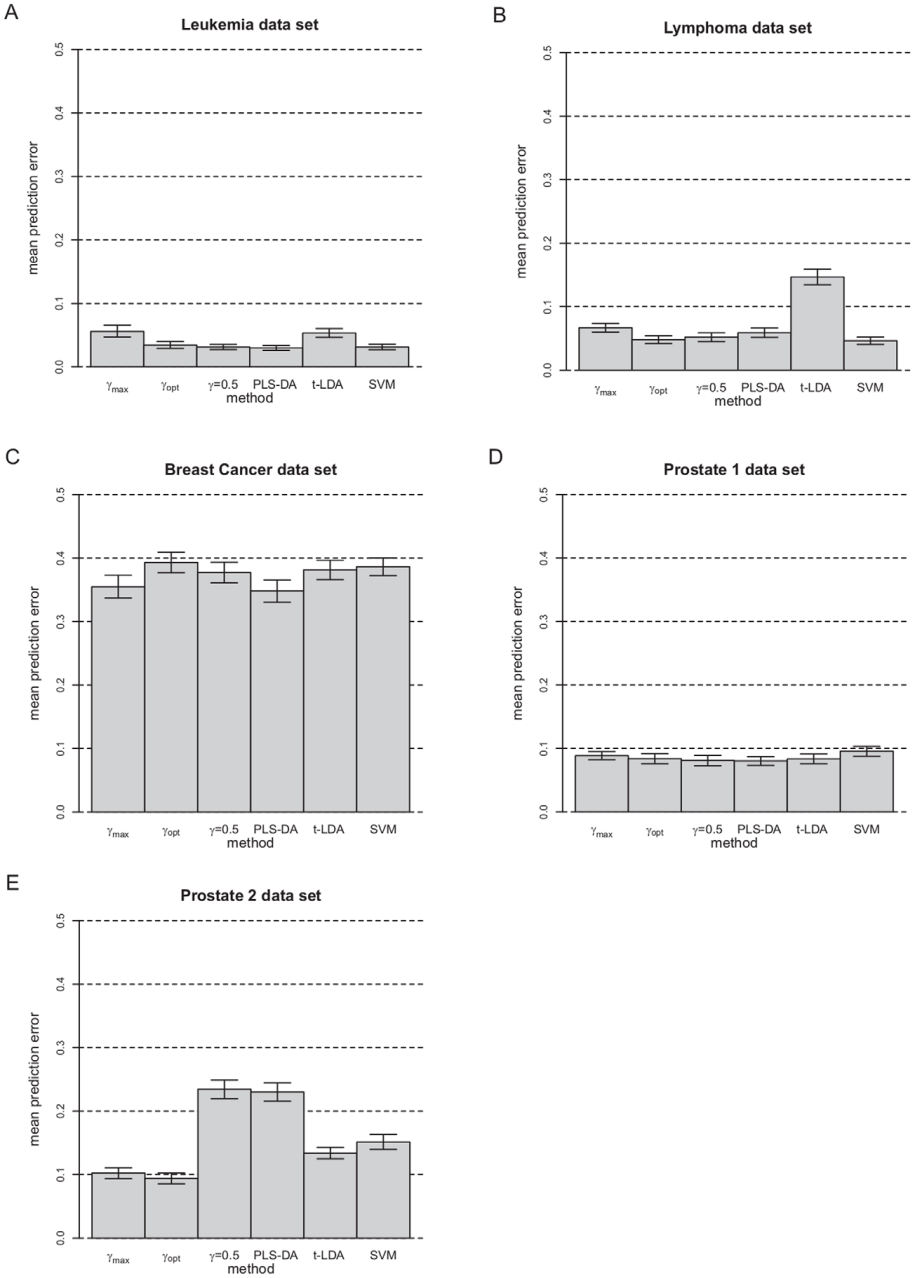
Mean PE of PPLS-DA using 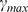, 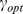 and 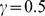, PLS-DA, t-LDA and SVM for the five cases of the experimental data sets.

In Figure 7 the mean PE results and the corresponding 95% confidence intervals are shown for all five experimental data sets for all considered methods. For PPLS-DA using 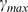, 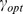 and 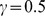 and PLS-DA, the average number of of components are shown in Table 3.

**Table 3:** The mean number of components used for the experimental data sets.

	PPLS-DA with			PLS-DA
data set	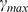	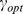	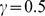	
Leukemia	2.6	2.8	2.3	2.0
Lymphoma	2.9	3.1	2.8	1.8
Breast cancer	2.4	2.3	2.4	2.7
Prostate 1	2.5	3.4	4.0	4.1
Prostate 2	2.9	3.7	4.3	4.2

Leukemia data set: For this data set, we found no significant differences in the PE of PPLS-DA using 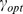 compared to PPLS-DA using 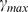 (Figure 7A), and both methods use similar numbers of components. The modal value of 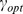 is 0.5, in comparison to 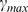 with two accumulations one around 0.3 and the other around 0.8 (see Figure S3, see Supplementary Materials). Comparing the above PE results to PLS-DA, then PLS-DA shows an equal PE compared to all four extensions of PPLS-DA. The PE of PPLS-DA using 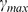 is significantly larger than the PE of PPLS-DA with 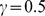 and PLS-DA. PLS-DA uses also in average the lowest number of components (2.0).

For PPLS-DA using 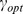 we find a significantly lower PE than for t-LDA, but for SVM the PE is similar to that of PPLS-DA using 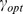.

Lymphoma data set: The PE of PPLS-DA using 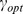 is significantly lower than the PE of PPLS-DA using 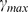 (Figure 7B). PPLS-DA using 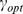 leads in average to 3.1 components and PPLS-DA using 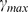 to 2.9 components.

Considering PLS-DA, we find a significantly lower PE than for PPLS-DA using 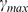, which is equal to the PEs of PPLS-DA using 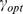 and 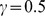. PLS-DA used the smallest average number of components (1.8), for PPLS-DA using 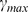 and 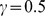 we find similar number of components in average 2.9 and 2.8.

The method t-LDA shows a significantly higher PE in comparison to PPLS-DA using 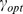, and SVM an equal PE to PPLS-DA using 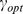.

Breast cancer data set: For this data set, PPLS-DA using 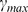 and PPLS-DA using 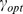 does not show significantly different PEs (Figure 7C). The number of components used are also similar for these two methods with 2.4 for PPLS-DA using 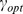 and 2.3 for PPLS-DA using 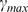.

Also PLS-DA shows an equal PE and a slightly higher number of components (2.7), compared to PPLS-DA using 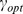 and 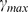 or 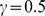.

If compared to PPLS-DA using 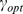, t-LDA and SVM show similar PE.

Prostate 1 data set: PPLS-DA using 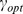 shows equal PE to PPLS-DA using 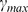 (Figure 7D). PPLS-DA using 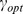 leads in average to 3.4 components, while PPLS-DA using 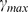 uses 2.5 components.

Investigating PLS-DA, the PE is equal to the PE of PPLS-DA using 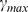 or 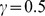. PPLS-DA using 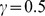 and PLS-DA use the largest average number of components, 4.1 and 4 (see Figure S4).

Also t-LDA and SVM show equal PEs to the other methods.

Prostate 2 data set: The PE of PPLS-DA using 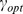 is equal to the PE of PPLS-DA with 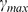 (Figure 7E). Moreover, PPLS-DA using 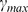 used 2.9 components and PPLS-DA using 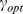 uses 3.7 components.

PPLS-DA using 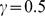 and PLS-DA show equal PE and significantly higher PE than for PPLS-DA using 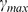 or using 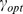. PPLS-DA with 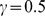 and PLS-DA use more components in comparison to all versions of PPLS-DA.

Both methods t-LDA and SVM show a significantly larger PE than PPLS-DA using 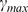 or PPLS-DA using 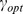, but a significantly lower PE than PLS-DA and PPLS-DA using 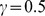.

## Discussion

The focus of our study is on introduction of an extension for the method PPLS-DA for better classification in high-dimensional datasets, as for example gene expression datasets in biomedicine. The optimization criterion for the power parameter 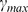 in the ordinary PPLS-DA is towards canonical correlation, and does not need to be best for prediction. Our extension of PPLS-DA introduces optimization of 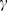 with respect to prediction using an inner cross-validation approach. We carry along comparisons to LDA and SVM, to bring our proposed method into line with these standard classification methods.

### Comparison between PPLS-DA using 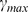 and 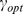


The PEs of the outer test sets for PPLS-DA were improved or showed at least equal values by optimization of the 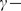
value with respect to the prediction with LDA in comparison to PPLS-DA using 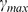, for all simulated data and the experimental data sets.

#### Simulated data

Comparing the histograms of 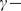
values found by PPLS-DA using 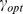 and PPLS-DA with 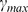, the reason for the lower PE can be traced back to the down-weighting of the non-informative features for the simulated data (see Figure S2 and Figure 6). The loading weights for these features are near or equal to zero. The influence of the features, which are not informative for the discrimination (and can be interpreted as noise), is reduced, because the impact on the calculation of the components is lower for PPLS-DA using 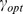 than for PPLS-DA with 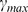. Values of 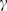 near to one, leads to preference of features which show a high correlation to the dummy response (in the simulation study these are the differentially expressed genes). Changing the optimization criterion from correlation towards prediction, leads also to a lower average number of components for the simulated data.

#### Experimental data

For the experimental data, the 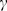-values determined by the canonical correlation (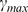) are larger than the 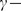
values detected by our proposed extension of PPLS-DA. Even if our analyses of simulated data suggested lower PE values for larger choices of 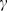, for the Leukemia data set and the Lymphoma data set also PPLS-DA using 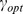 shows a significantly lower PE than PPLS-DA using 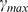. Note, that the true informative genes for the experimental data are not known, and the proportion of differentially expressed genes most likely is much larger than for the simulated data, therefore the comparison of the results for simulated and experimental data is not straightforward. Moreover experimental data are usually noisier than simulated data. The part of reality we have not been able to model in the simulated data might be a sort of noise and data structure that we cannot improve on, regardless our choice of 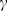. If we could remove this part of the noise from the real data, the relative improvements might be just as good as with the simulated data.

Summarizing the findings for the simulated data, PPLS-DA using 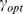 shows significantly lower PEs than PPLS-DA with 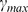. For the experimental data, the results are also significantly lower or equal for the extensions considering the PE.

Additionally we had considered three further versions to determine the power parameter towards prediction. These versions for example differ in the number of considered components for the optimization of the power parameter and also one version which optimizes an individual power parameter for each component was studied. The results are similar to those of the extension presented here (data not shown).

### Comparison between PPLS-DA using 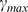 and PLS-DA

The development of PPLS-DA followed the development of powered partial least squares as a natural extension of the power methodology to handle discrete responses. Several factors motivated this advancement to PLS-DA. First the application of powers enables focusing on fewer explanatory variables in the loading weights, smoothing over some of the noise in the remaining variables. Second, focus can be shifted between the correlation and standard deviation parts of the loading weights, which is even more important for discrete responses. Finally, the maximization criterion is moved from the between-group variation (B) to the product of the between group variation matrix and the inverse of the within-group variation matrix (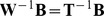). This has the effect of moving from covariance maximization to a correlation maximization. Instead of just searching for the space having highest variation between the groups, we also minimize the variation inside the groups, increasing the likeliness of good group separation.

In our study, PPLS-DA with 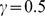 (applying no power parameter) and PLS-DA always show equal PEs for the simulated and the experimental data sets. Hence, for this case the different optimization tasks show no great differences with respect to the PEs of the outer test sets. Including the power parameter, the PE of PPLS-DA using 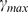 is equal to the PE of PLS-DA for all simulated data. Also, the number of components used is in average lower or equal for PPLS-DA using 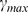 than for PLS-DA.

For two of the five experimental data sets (Leukemia and Lymphoma), the PE of PPLS-DA using 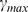 is significantly higher than the PE of PLS-DA. For these data sets, the proportions of differentially expressed genes are large (24.4% and 40.5%) and the genes are only weakly linear dependent (considering the condition indexes). The PE of PPLS-DA using 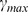 is significantly lower than for PLS-DA for the Prostate 2 data set, and the number of components used is also in average lower. This data set contains only a low proportion of 1.4% differentially expressed genes, and the total number of genes is very high (42129). Moreover, for this data set, the genes show a stronger linear dependency (rapid increase of the condidion index) than for the Leukemia or the Lymphoma data set.

Summarizing, a weak increase of 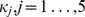 indicates no improvement for the PE when using PPLS-DA with 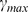 instead of PLS-DA. Concerning percentage of DEGs, the PE of PPLS-DA using 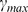 is equal to the PE of PLS-DA only for a small percentage of DEGs (Breast Cancer data and case 3 of the simulated data). For a weak increase of 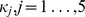 and high percentage of DEGs, the PE for PPLS-DA using 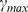 is even larger than for PLS-DA (Leukemia and Lymphoma data sets).

A rapid increase of 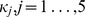 and a large proportion of DEGs, using 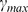 instead of PLS-DA does not improve the PE (Prostate 1). On the contrary, for a rapid increase of 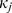 and the case of small percentage of DEGs, we can improve the PE, employing PPLS-DA using 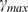 instead of PLS-DA (Prostate 2).

### Comparison between PLS-DA and PPLS-DA using 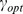


For the simulated data, PLS-DA shows equal PE if compared to PPLS-DA using 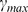 or 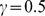. The PE of PPLS-DA using 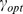 is significantly lower than the PE of PLS-DA. For the experimental data, for four of the five data sets (Leukemia, Lymphoma, Breast Cancer and Prostate 1), the PEs of PLS-DA and PPLS-DA using 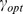 show no significant differences. For the Prostate 2 data set, the PE of PPLS-DA using 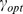 is clearly lower than for PLS-DA.

We conclude first, equal PEs between PLS-DA and PPLS-DA using 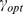 are caused by a weak between-feature dependency, independent of the proportion of DEGs. Second, a data set with strong collinearity between the features and a low number of DEGs, in contrary shows a clearly lower PE for PPLS-DA using 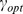 than for PLS-DA.

### Comparison between PPLS-DA using 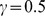 and PLS-DA

The PEs for PLS-DA and PPLS-DA using 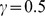 are non-distinguishable for all simulated and all experimental data sets investigated. Maximization of the covariance or maximization of the correlation without the power parameter, results in equal PEs of the outer test set.

### Comparison between PPLS-DA using 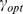 and t-LDA and SVM

The more classic and widely available classification methods t-LDA and SVM were also run and compared to PPLS-DA using 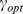 on all simulated and experimental data sets. For the simulated data, t-LDA performs indistinguishable well as PPLS-DA using 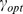. For the experimental datasets, SVM draws level with our proposed approach except for the case of Prostate 2. In this comparison, PPLS-DA using 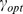 shows a comparatively stable well performance. There may, however, exist further statistical learning methods which outperform the methods presented in this study. t-LDA and SVM should serve to demonstrate comparability of PLS-related methods to other commonly chosen approaches for the classification problems. The focus of our study, however, was on further developing PPLS-DA, a so-called multivariate method, which has already been proven its large potential for classification problems involving magnitude more features than samples as it is the case in OMICs data sets.

### Conclusions and Outlook

It is conceivable to use results of an initial PPLS-DA cross-validation series, optimizing 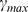, to try to judge if running the extended version would be rewarding. Data sets with a high proportion of differentially expressed genes and weak linear dependency (like the Leukemia data set and the Lymphoma data set) most probably show good prediction results for PLS-DA. Here, we found no gain using PPLS-DA with powers (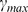 or 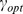). On the contrary, for a rapid increase of the condition index, a low proportion of differentially expressed genes and a large total number of genes, using PPLS-DA with 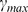 clearly improves the prediction error compared to PLS-DA. In cases where PPLS-DA using 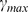 gives no advantages over PLS-DA, using the extensions of PPLS-DA (optimizing the power parameter) for prediction can be advantageous. Starting to analyse the eigenvalue structure and the number of differentially expressed genes, can possibly be useful to decide which method to use. One aspect of future work is to validate our conclusions by additional experimental data sets as well as further simulations implementing a more complex covariance structure.

## Supporting Information

Figure S1Mean PE of PPLS-DA for simulated data using 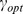 plotted against 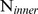. The simulated data of case 3 are constructed such that the technical variance is of the same size as the biological variance. 10 differentially expressed genes with a mean class difference 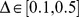 are simulated. For different numbers of the cross-validation steps, the mean prediction error (PE) and the corresponding 95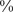 confidence intervals are shown for PPLS-DA using 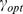 for the determination of the power parameter. Two stepsizes 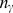 are considered for the fragmentation of the interval [0,1], 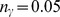 (A) and 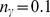 (B). The basis are the results of 100 choices of the outer training and outer test set.(TIFF)

Figure S2Histograms of 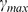 and 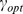 for the simulated data (case 3). The simulated data of case 1 are constructed such that the technical variance is of the same size as the biological variance. 10 differentially expressed genes with a mean class difference 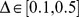 are simulated. Values of 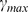 detected by PPLS-DA for the first component (A) and for all components (B). In panel (C) the 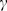-values are shown, detected for 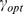 with 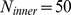 and stepsize 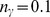. The basis are the results of 100 choices of the outer training set.(TIFF)

Figure S3Histograms of 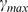 and 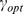 for the Leukemia data set. Values of 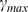 detected by PPLS-DA for the first component (A) and for all components (B). In panel (C) the 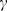-values are shown, detected for 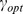 with 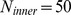 and step size 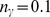. The basis are the results of 100 choices of the outer training set.(TIFF)

Figure S4Histograms of 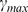 and 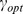 for the Prostate 1 data set. Values of 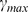 detected by PPLS-DA for the first component (A) and for all components (B). In panel (C) the 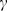-values are shown, detected for 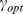 with 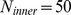 and step size 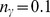. The basis are the results of 100 choices of the outer training set.(TIFF)

## References

[1] Wold S, Albano C, Dunn II W, Esbensen K, Hellberg S, et al.. (1983) Pattern recognition: Finding and using regularites in multivariate data, applied Sciences publ. London. 147–188.

[2] Martens SWH, Wold H (1983) The multivariate calibration problem in chemistry solved by the pls method. Proc Conf Matrix Pencils (ARuhe, BKaagstroem, eds) March 1982 Lecture notes in Mathematics : 286–293.

[3] Barker M, Rayens W (2003) Partial least squares for discrimination. Journal of Chemometrics 17(3): 166–173.

[4] Nocairi H, Qannari EM, Vigneau E, Bertrand D (2005) Discrimination on latent components with respect to patterns. application to multicollinear data. Computational Statistics & Data Analysis 48 (1): 139–147.

[5] Liland KH, Indahl U (2009) Powered partial least squares discriminant analysis. Journal of Chemometrics 23: 7–18.

[6] Indahl UG (2005) A twist to partial least squares regression. Journal of Chemometrics 19: 32–44.

[7] Telaar A, Repsilber D, Nürnberg G (2011) Biomarker Discovery: Classification using pooled samples - a simulation study. Computational Statistics.

[8] Indahl UG, Martens H, Næs T (2007) From dummy regression to prior probabilities in pls-da. Journal of Chemometrics 21: 529–536.

[9] Indahl UG, Liland KH, Næs T (2009) Canonical partial least squares - a unified pls approach to classification and regression problems. Journal of Chemometrics 23: 495–504.

[10] Dimitriadou E, Hornik K, Leisch F, Meyer D, Weingessel A (2009) e1071: Misc functions of the department of statistics (e1071). TU Wien.

[11] Dettling M (2004) Bagboosting for tumor classification with gene expression data. Bioinformatics 20: 3583–3593. 15466910 10.1093/bioinformatics/bth447

[12] Sæbø S, Almøy T, Aarøe J, Aastveit AH (2008) ST-PLS: a multi-directional nearest shrunken centroid type classifier via PLS. Journal of Chemometrics 20: 54–62.

[13] Belsley DA, Kuh E, Welsch RE (1980) Regression Diagnostics: Identifying Inuential Data and Sources of Collinearity. John Wiley & Sons.

[14] Helland IS, Almøy T (1994) Comparison of prediction methods when only a few components are relevant. Journal of the American Statistical Association 89: 583–591.

[15] Storey J, Tibshirani R (2003) Statistical signilficance for genomewide studies. Proc Natal Acad Sci 100: 9440–5. 10.1073/pnas.1530509100 PMC170937 12883005

[16] Dudoit S, Fridlyand J, Speed TP (2002) Comparison of discrimination methods for the classification of tumors using gene expression data. Journal of the American Statistical Association 97: 77–87.

[17] van’t Veer LJ, Dai H, van de Vijver MJ, He YD, Hart AA, et al (2002) Gene expression profiling predicts clinical outcome of breast cancer. Nature 415: 530–536. 11823860 10.1038/415530a

[18] Dettling M, Buehlmann P (2003) Boosting for tumor classification with gene expression data. Bioinformatics : 1061–1069. 10.1093/bioinformatics/btf867 12801866

[19] Golub TR, Slonim DK, Tamayo P, Huard C, Gaasenbeek M, et al (1999) Molecular classification of cancer: class discovery and class prediction by gene expression monitoring. Science 286: 531–537. 10521349 10.1126/science.286.5439.531

[20] Shipp MA, Ross KN, Tamayo P, Weng AP, Kutok JL, et al (2002) Diffuse large b-cell lymphoma outcome prediction by gene-expression profiling and supervised machine learning. Nat Med 8: 68–74. 11786909 10.1038/nm0102-68

[21] Singh D, Febbo PG, Ross K, Jackson DG, Manola J, et al (2002) Gene expression correlates of clinical prostate cancer behavior. Cancer Cell 1: 203–209. 12086878 10.1016/s1535-6108(02)00030-2

[22] Lapointe J, Li C, Higgins JP, van de Rijn M, Bair E, et al (2004) Gene expression profiling identifies clinically relevant subtypes of prostate cancer. Proc Natl Acad Sci U S A 101: 811–816. 14711987 10.1073/pnas.0304146101 PMC321763

