# Supplementary figures and images for: An Extension of PPLS-DA for Classification and Comparison to Ordinary PLS-DA
Source: PLoS One. 2013 Feb 11;8(2):e55267. doi: 10.1371/journal.pone.0055267 (PMC3569448; doi:10.1371/journal.pone.0055267)

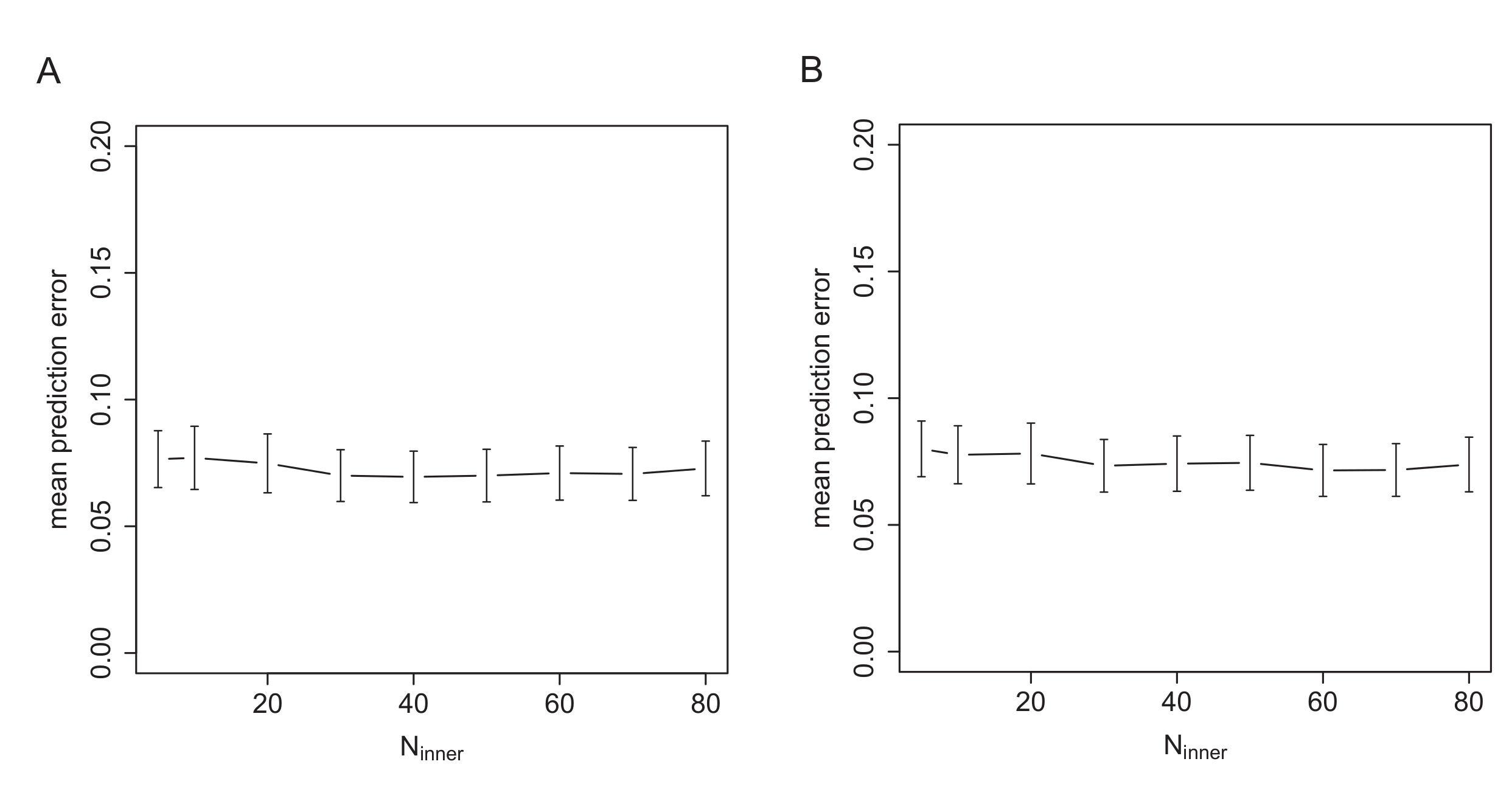

Supplement: Figure S1 — Mean PE of PPLS-DA for simulated data using plotted against . The simulated data of case 3 are constructed such that the technical variance is of the same size as the biological variance. 10 differentially expressed genes with a mean class difference are simulated. For different numbers of the cross-validation steps, the mean prediction error (PE) and the corresponding 95 confidence intervals are shown for PPLS-DA using for the determination of the power parameter. Two stepsizes are considered for the fragmentation of the interval [0,1], (A) and (B). The basis are the results of 100 choices of the outer training and outer test set. (TIFF) [file pone.0055267.s001.tiff]

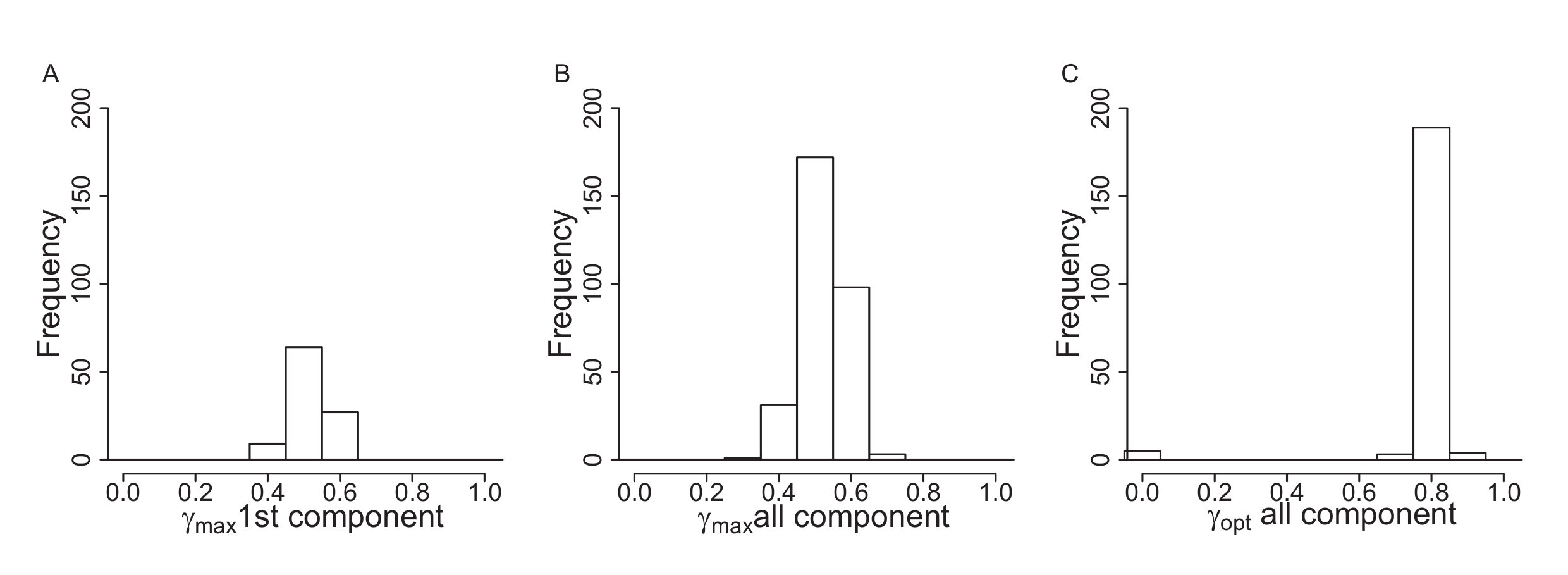

Supplement: Figure S2 — Histograms of and for the simulated data (case 3). The simulated data of case 1 are constructed such that the technical variance is of the same size as the biological variance. 10 differentially expressed genes with a mean class difference are simulated. Values of detected by PPLS-DA for the first component (A) and for all components (B). In panel (C) the -values are shown, detected for with and stepsize . The basis are the results of 100 choices of the outer training set. (TIFF) [file pone.0055267.s002.tiff]

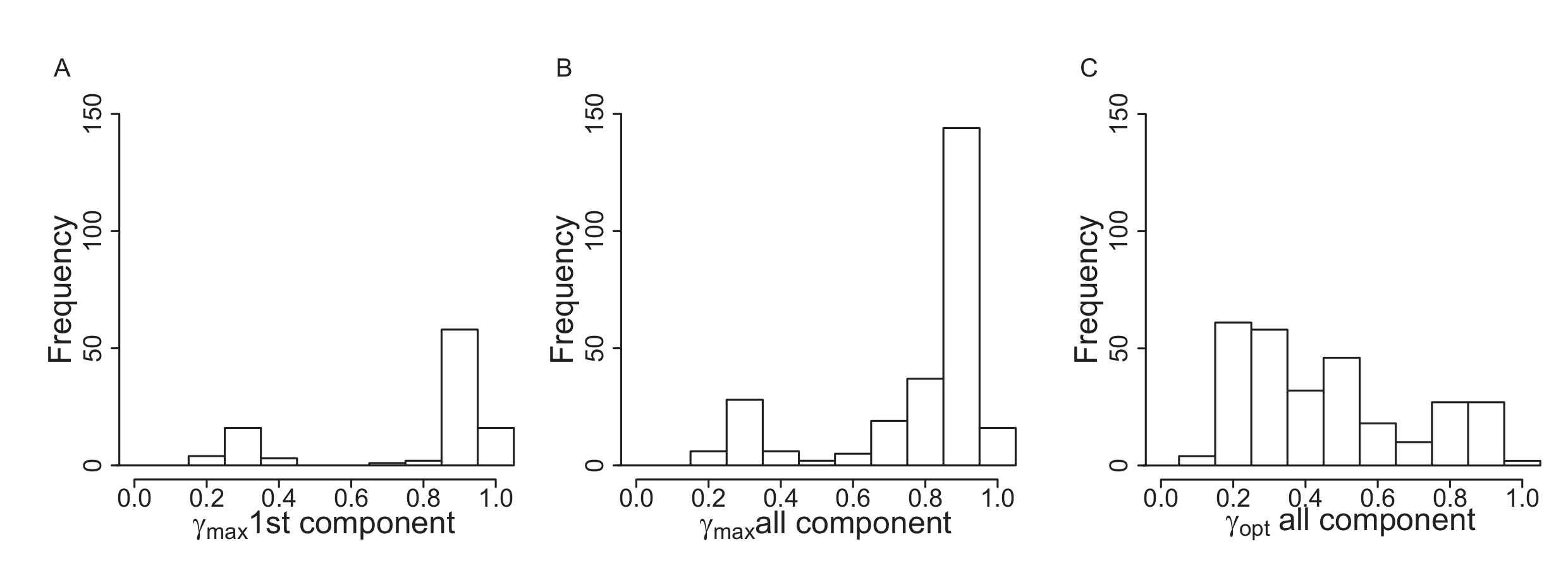

Supplement: Figure S3 — Histograms of and for the Leukemia data set. Values of detected by PPLS-DA for the first component (A) and for all components (B). In panel (C) the -values are shown, detected for with and step size . The basis are the results of 100 choices of the outer training set. (TIFF) [file pone.0055267.s003.tiff]

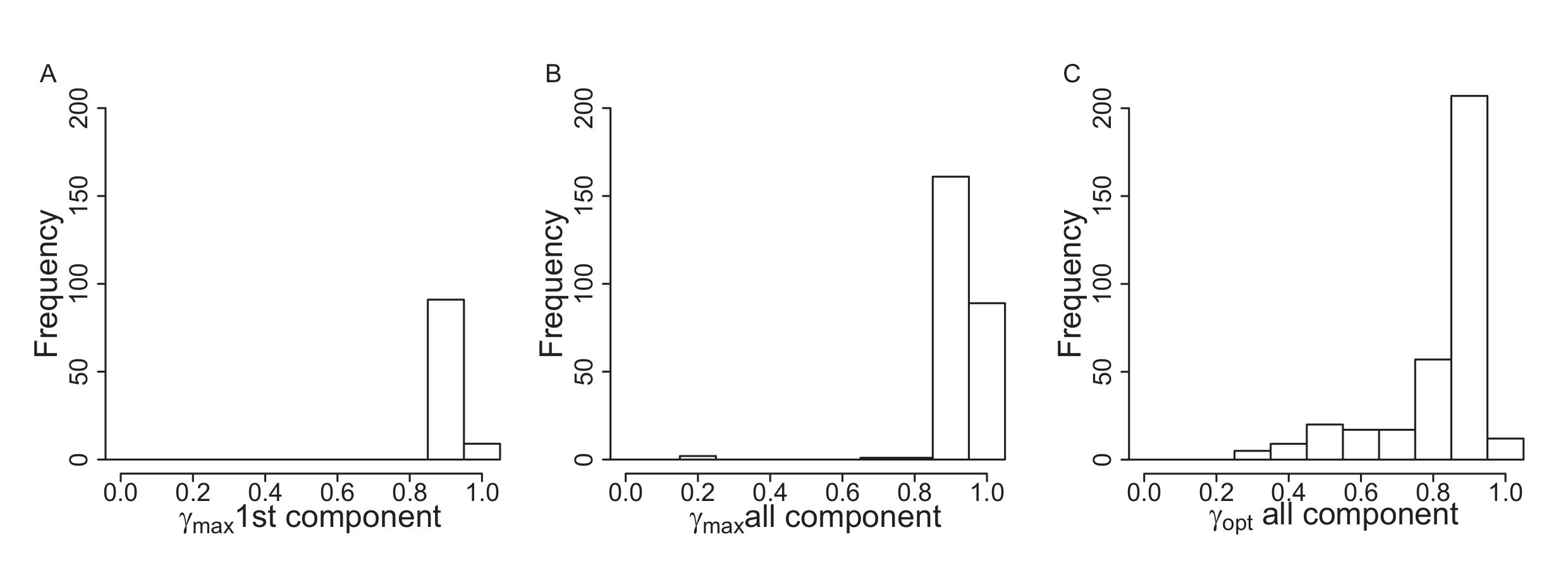

Supplement: Figure S4 — Histograms of and for the Prostate 1 data set. Values of detected by PPLS-DA for the first component (A) and for all components (B). In panel (C) the -values are shown, detected for with and step size . The basis are the results of 100 choices of the outer training set. (TIFF) [file pone.0055267.s004.tiff]
